# QuilA® adjuvanted Coxevac® sustains Th1-CD8^+^-type immunity and increases protection in *Coxiella burnetii*-challenged goats

**DOI:** 10.1038/s41541-023-00607-z

**Published:** 2023-02-14

**Authors:** Sara Tomaiuolo, Wiebke Jansen, Susana Soares Martins, Bert Devriendt, Eric Cox, Marcella Mori

**Affiliations:** 1 Bacterial Zoonoses Unit, Veterinary Bacteriology, Infectious Diseases in Animals Scientific Directorate, Sciensano, Brussels, Belgium; 2 National Reference Centre for Coxiella burnetii and Bartonella, Brussels, Belgium; 3 Laboratory of Immunology, Department of Translational Physiology, Infectiology and Public Health, Faculty of Veterinary Medicine, Ghent University, Merelbeke, Belgium

**Keywords:** Adjuvants, Bacterial host response

## Abstract

Coxevac® is the EMA-approved veterinary vaccine for the protection of cattle and goats against Q fever, a zoonotic bacterial disease due to *Coxiella burnetii*. Since Coxevac® reduces bacterial shedding and clinical symptoms but does not prevent infection, novel, ready-to-use vaccine formulations are needed to increase its immunogenicity. Here, a goat vaccination-challenge model was used to evaluate the impact of the commercially available saponin-based QuilA® adjuvant on Coxevac® immunity. Upon challenge, the QuilA®-Coxevac® group showed a stronger immune response reflected in a higher magnitude of total IgG and an increase in circulating and splenic CD8^+^ T-cells compared to the Coxevac® and challenged-control groups. The QuilA®-Coxevac® group was characterized by a targeted Th1-type response (*IFNγ*, *IP10*) associated with increased transcripts of CD8^+^ and NK cells in spleens and γδ T cells in bronchial lymph nodes. Coxevac® vaccinated animals presented an intermediate expression of Th1-related genes, while the challenged-control group showed an immune response characterized by pro-inflammatory (*IL1β*, *TNFα*, *IL12*), Th2 (*IL4* and *IL13*), Th17 (*IL17A*) and other immunoregulatory cytokines (*IL6*, *IL10*). An intriguing role was observed for γδ T cells, which were of TBX21- and SOX4-types in the QuilA®-Coxevac® and challenged control group, respectively. Overall, the addition of QuilA® resulted in a sustained Th1-type activation associated with an increased vaccine-induced bacterial clearance of 33.3% as compared to Coxevac® only. QuilA® could be proposed as a readily-applied veterinary solution to improve Coxevac® efficacy against *C. burnetii* infection in field settings.

## Introduction

Q fever is a zoonotic bacterial infection leading to significant public health and economic issues worldwide. The causative agent, C*oxiella burnetii*, is a highly infectious intracellular Gram-negative bacterium, which is extremely resistant in the environment^1^. In ruminants, *C. burnetii* infection causes abortion, stillbirth and infertility, but distinct clinical outcomes are observed between ruminant species: abortion storms in goats and sheep, and sporadic abortion cases in cattle^2,3^. The parturition or immediate postpartum periods are those at the highest risk in terms of bacterial shedding, however, the excretion might occur also beyond these periods by clinically affected or asymptomatic animals^4,5^.

Knowledge about the pathogenesis of *C. burnetii* and the associated immune responses in domestic ruminants is scarce. In pregnant goats, bacterial replication starts in the trophoblasts of the allantochorion^6–8^. Bacterial DNA can be detected in the placenta 4 weeks post-infection (wpi), suggesting that *C. burnetii* needs time to multiply until reaching detectable levels^8^. Massive bacterial multiplication occurs however in the last weeks of gestation, before abortion^7^. From 6 wpi, *C. burnetii* DNA can also be present in other organs than the placenta^8^. Whether the function of these infected organs is affected by the presence of *C. burnetii* is yet unknown.

Infection of goats with *C. burnetii* induces considerable antibody responses, indicating that humoral immunity contributes to the defense mechanisms against Q fever. A similar magnitude of antibody responses has been observed in pregnant and non-pregnant goats^9,10^. In both, IgM and IgG antibodies against *C. burnetii* phase II (phII, avirulent) antigens appear from 3 wpi onwards. In non-pregnant goats, phase I (phI, virulent) IgM and IgG titers increase after 3 and 4 wpi, respectively. This anti-phI antibody response is delayed in infected pregnant goats.

Although cell-mediated immune responses are important for protection against infection with intracellular pathogens, both the nature of the induced cell-mediated immune responses and their role in protecting goats from infection with *C. burnetii* remain poorly described. Roest et al.^9^ showed that cytokine mRNA levels in peripheral blood of pregnant goats increased before (*IL10*) or after (*TNFα, IL1β*) parturition. This coincided with an up-regulation of the total IFNγ production in peripheral blood mononuclear cells (PBMCs) at one-week post parturition^9^. Ammerdorffer et al.^11^ also observed increased *IFNγ* and *TNFα* transcript levels in antigen-stimulated PBMCs from infected pregnant goats. Thus, although some studies reported changes in cytokine production by PBMCs in pregnant goats, the identity of the involved cells remains unknown. In addition, no information is available regarding non-pregnant goats.

A deeper understanding of the pathogenesis of *C. burnetii* and the immune responses raised upon infection of domestic ruminants may contribute to the redefinement and the improvement of ruminant vaccines, deployed as a preventive measure to limit Q fever in animals and halt transmission to humans. In Europe, the Coxevac® non-adjuvanted whole-cell formalin-inactivated ph I vaccine (Ceva Santé Animale, Libourne, France) is used to protect cattle and goats against Q fever^12^. Although several studies demonstrated its efficacy in reducing clinical signs (abortions) and bacterial shedding^13–16^, this vaccine does not prevent infection^13–15^ nor clears the infection in infected animals^17,18^. In addition, field data indicated that antibody levels decrease after 9 months below protective levels, hampering targeted annual vaccination programs on herd level^19^. If the critical level of protective immunity is not maintained long enough at the individual or herd level, recrudescence will likely take place^19,20^. In this case, consistent control of *C. burnetii* infection in ruminants is only reached through the use of costly and multiannual compliant vaccination protocols^14,19^.

Many vaccines require the addition of adjuvants to induce an appropriate immune response for protection upon challenge. Adjuvants are able to increase the magnitude of the vaccine induced-immune responses, and, importantly, to tailor immune responses to each specific pathogen. Indeed, certain adjuvants can drive specific immune responses (i.e., CD4^+^ vs CD8^+^ T cells, Th1 vs Th2 response, distinct antibody isotypes), encourage immune memory responses or activate a quicker initial response^21^. QuilA®, a saponin adjuvant extracted from *Quillaja saponaria*, is widely used in veterinary vaccines due to its capacity to stimulate both CD4^+^ helper and CD8^+^ cytotoxic cells as well as to induce long-lasting antibody responses^22–27^. It is an EMA-approved veterinary medicinal product and therefore immediately applicable in field settings. Considering both the non-adjuvanted nature of Coxevac® and the QuilA®-driven induction of T cell immunity, we selected this adjuvant as a candidate for improving the efficacy and immunogenicity of Coxevac®.

In this study, we characterized the immune response induced by Coxevac® vaccination and evaluated the influence of QuilA® on vaccine immunogenicity and efficacy in goats. In order to mimic natural infections, goats were challenged for the first time with an aerosolized axenic culture of *C. burnetii*, isolated from an infected goat in 2010. Considering the intracellular nature of the bacterium, obtaining an axenic culture from field samples is extremely rare and challenging. Our data demonstrated that Coxevac® did not generate an immune response able to confer substantial protection upon challenge. However, the addition of the QuilA® adjuvant enhanced the humoral response and induced a sustained Th1-type cellular response resulting in increased vaccine efficacy.

## Results

**Fig. 1:**
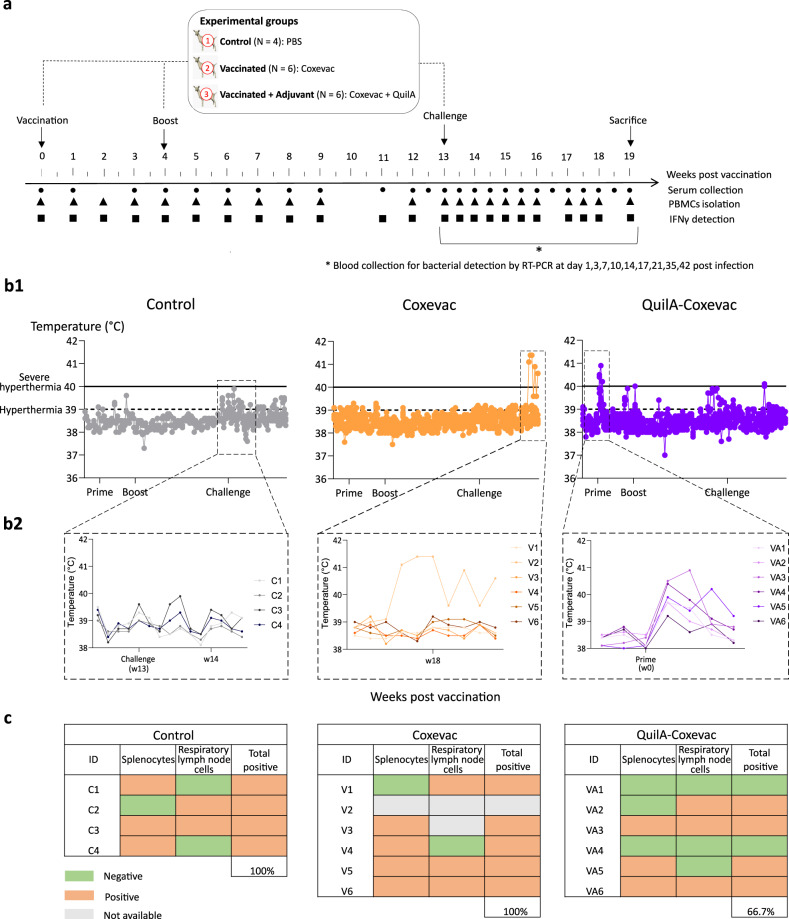
Overview of the experimental design, daily temperature and bacterial burden of the vaccination-challenge experiment in goats. **a** A prime–boost strategy was used for the vaccination of goats with Coxevac® (*n* = 6) or QuilA-Coxevac® (*n* = 6). As controls, goats (*n* = 4) were similarly injected with PBS. At week 13 post vaccination, all groups were intranasally challenged with the *C. burnetii* CbBEC2 strain. At the indicated time points, serum was collected to evaluate the specific antibody response, PBMCs (peripheral blood mononuclear cells) were isolated for T and B cell phenotyping and the interferonγ (IFNγ) production was detected in antigen stimulated PBMCs. At sacrifice, organs were collected for bacterial detection, cell phenotyping and gene expression profiling. **b** QuilA®-Coxevac® prime vaccination induces a transient increase in the rectal temperature. **b1** Daily monitoring of rectal temperature upon vaccination and challenge for the control, Coxevac® and QuilA®-Coxevac® groups. **b2** Zoom on selected time points for each group to display specific patterns of goats. Individual daily values are represented for all animals (**b1**,**2**). **c** *C. burnetii* detection by PCR assay in splenocytes and bronchial lymph node cells isolated at sacrifice. Samples were injected in embryonated eggs (in triplicates) for bacterial amplification and only yolk sacs of eggs dying after day 5 post injection were collected. V2 splenocytes and V2 and V3 respiratory lymph node cells were highly contaminated and amplification in eggs was not possible. Goats presenting at least one positive result per organ were considered to be positive. W = week after vaccination, C = control goat, V = vaccinated goat (Coxevac®), VA = vaccinated plus adjuvant goat (QuilA®-Coxevac®).

Effective action of Coxevac® is usually achieved following compliant multiannual vaccination protocols, but field data indicated that protective antibody levels are not maintained long enough to establish standardized yearly vaccination programs^19^. Given that Coxevac® does not contain an adjuvant and the need to increase its efficacy, we designed an improved formulation of Coxevac® by including the ready-to-use QuilA® adjuvant in the emulsion. This new formulation was assessed in a vaccination-challenge experiment using Saanen goats and inocula prepared from axenic cultures of the CbBEC2 strain (P6) (Fig. 1a).

### In vitro and in vivo quality controls of the bacterial inoculum

The CbBEC2 strain was previously isolated in axenic medium at the National Reference Centre (Sciensano, Belgium). As the infective capacity of *C. burnetii* strains is determined by the expression of a phI lipopolysaccharide (LPS)^28^, we verified the presence of a full-length LPS in this strain. The existence of genes involved in the synthesis of a phI LPS was investigated by Whole Genome Sequencing (WGS). The alignment of the CbBEC2 assembly and the Nine Mile (NM) phI DNA sequence (encoding for the operon involved in the biosynthesis of the complete LPS) resulted in 99.92% similarity, indicating an operon genetic integrity in the CbBEC2 strain. The complete LPS sequence was localized in contig 11 as illustrated in Supplementary Fig. 1a. To further examine the phase properties of the LPS, CbBEC2 at passage 6 (P6) was cultivated for 14 days in Acidified Citrate Cysteine Medium-2 (ACCM-2) and LPS was isolated and profiled on a silver-stained gel (Supplementary Fig. 1b). The phI pattern of CbBEC2 LPS was unique and differed from that of NM phI LPS (bands above 10 kDa). As LPS was isolated from the CbBEC2 strain at P6, we observed co-appearance of intermediate (around 10 kDa) and minor presence of phII (far below 10 kDa) LPS forms. These LPS features could be attributed to the serial passages^29^ or the intrinsic nature of the strain^30^. To verify that the infectious potential of the CbBEC2 axenic cultures (P6) was retained after passaging, we compared the proliferative capacity of CbBEC2 from freshly collected splenic harvest at P2 (estimated m.o.i. of 5 × 10^3 ^G.E.) to that from the axenic culture at P6 (estimated m.o.i. of 4 × 10^3 ^G.E.) in a mouse model. The axenic culture at P6 triggered significantly higher bacterial loads than the P2 in the spleen (Supplementary Fig. 1c, mean week1–week4 of 7.7 × 10^5 ^G.E./g vs 5.3 × 10^4 ^G.E./g). Also, the axenic strain triggered significantly higher organ weights in the spleen and liver and significantly higher IgM and IgG levels (Supplementary Fig. 2). Overall, these results validated the infectivity, the genetic features and the quality of the inoculum prepared from CbBEC2 cultures P6, which will be used in the next experiment for the challenge infection of goats.

### QuilA®-Coxevac® prime vaccination induces a transient increase of the rectal temperature

None of the control and the Coxevac® goats experienced severe hyperthermia (>40 °C), as indicated by the rectal temperature, at any time point after prime vaccination or boost (Fig. 1b1). One day after prime vaccination, all animals from the QuilA®-Coxevac® group presented a rise in the rectal temperature (average of 39.9 °C ± 0.5), with three goats experiencing severe hyperthermia on days 1 to 3 (Fig. 1b2). After challenge, no major changes were observed in the groups, although a slight increase of the average temperature was observed in the control animals a few days post-challenge. One goat (V2) of the Coxevac® group experienced severe hyperthermia from day 32 post-challenge towards the end of the experiment (Fig. 1b2) and presented clinical signs attributable to mastitis (the left mammary gland was hard, swollen, reddish and sensitive to touch).

### QuilA®-Coxevac® vaccination induced a robust and sustained anti-*C. burnetii* IgG response and an increased protection efficacy against Q fever

**Fig. 2:**
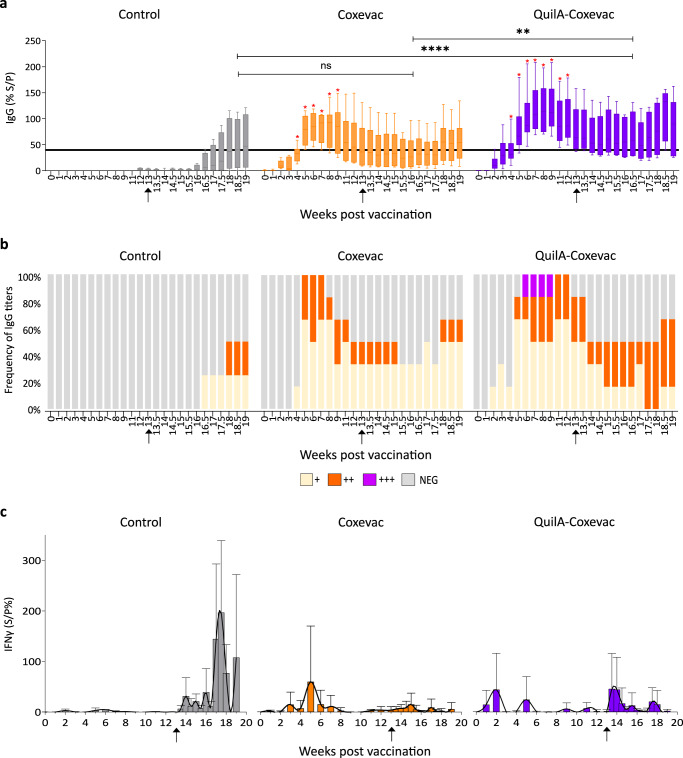
Kinetics of antigen specific-IgG and IFNɣ production upon vaccination and challenge infection. **a** QuilA®-Coxevac® vaccination induced robust and sustained *C. burnetii*-specific serum IgG titers compared to the Control and Coxevac® group. Boxplots represent IgG titers calculated as the percentage of sample/positive (S/P%) ratio for each sample. S/P% > 40 was considered as positive (values above the black line). The antibody response was analyzed after vaccination with a multiple unpaired T test with Welch correction followed by a two-stage linear step-up method of Benjamini, Krieger and Yekutieli to correct for multiple comparisons by False Discovery Rate (FDR) (red asterisks, *FDR ≤ 0.01). After challenge, differences between the three groups were assessed using the Kruskal–Wallis test with Dunn’s multiple comparison post hoc test (black asterisks, **p ≤ 0.01; ****p ≤ 0.0001). Boxplots are extended from the 25^th^ to 75^th^ percentiles, with a line at the median, and whiskers go from minima to maxima. **b** Frequency of anti-*C. burnetii* IgG titers for the control, Coxevac® and QuilA®-Coxevac® group. Neg = Titers ≤40; + = 40 < titers ≤ 100; ++ = 100 < titers ≤ 200; +++ = 200 < titers ≤ 300. **c** Differential IFNγ secretion by PBMCs stimulated with inactivated whole-cell *C. burnetii*. The IFNγ response was analyzed after challenge using mixed-effects models with Geisser–Greenhouse correction (data presented in the result section). Data are represented as mean ± SD. IFNγ secretion trends (in black) were visualized via interpolation of cubic splines. ↑ = moment of challenge.

After vaccination, IgG antibodies against *C. burnetii* were significantly more protracted in the QuilA®-Coxevac® than in the Coxevac® group (4 to 12 weeks post vaccination (wpv) vs 4 to 9 wpv) as opposed to the control goats (Fig. 2a). The first goat that reached levels above the threshold defined by the ELISA assay was detected at 2 wpv in goats vaccinated with QuilA®-Coxevac®, whilst only at 4 wpv in the Coxevac® goats (Fig. 2b). All goats from the Coxevac® and QuilA-Coxevac® groups exceeded the threshold after 5 and 6 wpv, respectively.

Upon challenge, the first goat of the control group that crossed the threshold level was detected at 16.5 wpv (3.5 weeks post-challenge, wpc) and an increase in the total IgG production was reached at 18 wpv (5 wpc) (Fig. 2b). Throughout the experiment, two control goats remained below the threshold defined by the ELISA assay. In the vaccinated groups, IgG levels rose again at 17.5 and 18 wpv in the Coxevac® and QuilA®-Coxevac® goats, respectively. After the challenge, the magnitude of the induced IgG antibodies was significantly higher in the QuilA®-Coxevac® group compared to control and Coxevac® animals (73% S/P vs 18% S/P and 43% S/P, respectively), while the control and Coxevac® group did not differ significantly in IgG levels (Fig. 2a).

As humoral immunity plays a role in the protection against Q fever^31–33^, we assessed whether vaccination with Coxevac® and QuilA®-Coxevac® was associated with increased protection. The presence of *C. burnetii* was investigated in several organs (*n* = 28) and blood after sacrifice (Fig. 1c and Supplementary Tables 1 and 2). The detection limit of the PCR assay was reached when results on DNA extracted from tissues were negative. Thus, splenocytes and bronchial lymph node cells were injected in embryonated eggs for bacterial amplification. An animal was considered positive if at least one of the two results was positive. *C. burnetii* was detected in 100% of the control and Coxevac® goats and in 66.7% of the QuilA®-Coxevac® goats (Fig. 1c) resulting in an increased vaccine-induced bacterial clearance of 33.3% as compared to Coxevac® only. Also, bacteremia was evaluated in all challenged goats at selected time points post-infection by PCR (Supplementary Table 2). In vaccinated goats, bacteremia was principally detected 1-day post-challenge. Taken together, these results confirmed that all goats were successfully infected with *C. burnetii* CbBEC2 strain and that QuilA® increased the Coxevac®-induced IgG response and protection.

### Differential IFNγ secretion profiles upon antigen-specific stimulation of PBMCs

To further explore cell-mediated immunity upon vaccination and challenge, we assessed the antigen-specific IFNγ response. PBMCs were ex vivo stimulated with *C. burnetii* antigens and the IFNγ production was measured at different time points. Upon vaccination, the IFNγ secretion profile in the Coxevac® group was detected as a unimodal response with a peak at 5 wpv (after the boost vaccine dose, 61% S/P). In contrast, the IFNγ kinetics of the QuilA®-Coxevac® group resulted in a bimodal response with the first peak of IFNγ production after the prime (2 wpv, 45% S/P) and the second peak after the booster (5 wpv, 25% S/P) vaccination (Fig. 2c). Upon challenge, completely different IFNγ patterns were observed for the three groups. In the control group, moderate levels of IFNγ production were detected immediately after the challenge (13.5 wpv), followed by a sudden increase at 17–17.5 wpv (4–4.5 wpc) and maintained until the end of the experiment. The IFNγ response of the Coxevac® group remained moderate and stable until the end of the experiment. In contrast, a bimodal trend characterized again the response of the QuilA®-Coxevac® group with the highest peak detected promptly after the challenge (13.5–14 wpv) and a second peak at 17.5–18 wpv. The mixed-effects model corroborated statistically significant differences between the three patterns post-challenge. This is illustrated by the interaction between time and group variables of control and Coxevac® (*F*(13, 102) = 5.02; *p* < 0.0001), control and QuilA-Coxevac® (*F*(13, 102) = 4.5; *p* < 0.0001), Coxevac® and QuilA®-Coxevac® (*F*(13, 129) = 1.96; *p* < 0.02) groups.

### The two Coxevac® formulations activate different immune cell subsets upon challenge

**Fig. 3:**
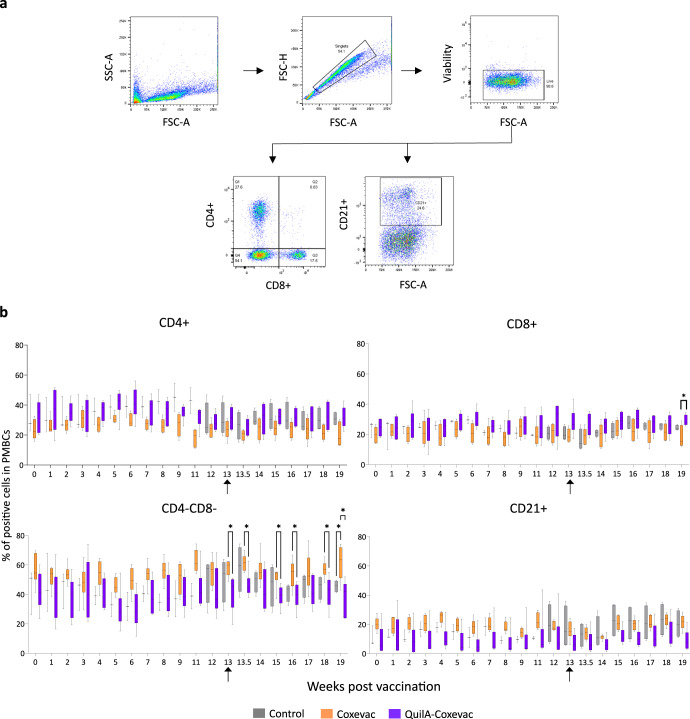
The two Coxevac® formulations activate cell subsets of different nature upon challenge. **a** Gating strategy used in the analysis of T and B lymphocytes in ex vivo stained PMBCs. Cellular subtypes were identified based on the expression of CD4, CD8 and CD21 cell markers. Plots are from a representative animal. Cell frequencies were calculated as percent of the viable cell population. **b** Kinetics of CD4^+^, CD8^+^, CD4^−^CD8^−^ and CD21^+^ cell frequencies in PBMCs upon vaccination and challenge. Each kinetic was analyzed using the mixed-effects models, both after vaccination and challenge, with Geisser–Greenhouse correction followed by Tukey’s multiple comparison post-hoc test (*p ≤ 0.05). Data are represented as boxplots extended from the 25^th^ to 75^th^ percentiles, with a line at the median, and whiskers go from minima to maxima. w = week after vaccination. ↑ = moment of challenge.

**Fig. 4:**
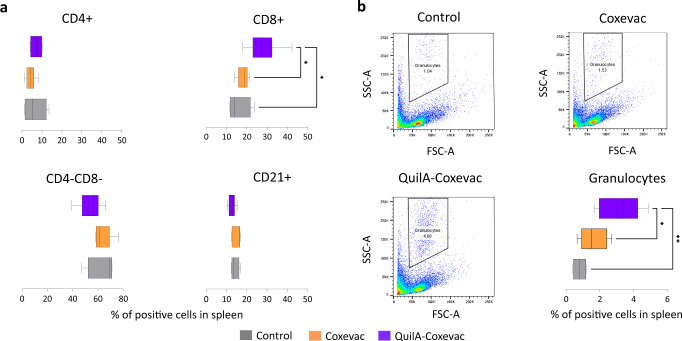
Higher frequencies of CD8^+^ cells and granulocytes detected in the spleen of the QuilA®-Coxevac® group at sacrifice. **a** Frequencies of CD4^+^, CD8^+^, CD4^−^CD8^−^ and CD21^+^ cells present in the spleen of control, Coxevac® and QuilA®-Coxevac® goats. The gating strategy is shown in Fig. 5a. **b** Gating strategy used in the analysis of granulocytes observed in goat spleens and quantification of their frequency. The granulocyte population was identified based on size and granularity characteristics. Group comparisons were performed using one-way ANOVA test with Tukey’s multiple comparison post-hoc test (*p ≤ 0.05; **p ≤ 0.01) (**a**, **b**). Cell frequencies were calculated as percent of the viable cell population and data are represented as boxplots extended from the 25^th^ to 75^th^ percentiles, with a line at the median, and whiskers go from minima to maxima (**a**, **b**).

Considering the differential IFNγ recall response observed in the three groups, we next assessed whether these differences could be attributed to distinctive activation of lymphocyte subpopulations. Therefore, we investigated the kinetics of T (CD4^+^ and CD8^+^) and B (CD21^+^) cells in PBMCs upon vaccination and challenge (Fig. 3a). The frequency of CD4^+^ and CD21^+^ cells did not change between groups after vaccination nor challenge (Fig. 3b). Likewise, vaccination did not influence the percentage of CD8^+^ cells detected before challenge, however, vaccinated groups showed significantly different patterns as compared to the control group after challenge. The results were validated with the mixed-effects model analysis that revealed a significant interaction between time and group variables of the control and Coxevac® (*F*(8, 64) = 2.48; *p* < 0.02) and control and QuilA®-Coxevac® (*F*(8, 64) = 2.68; *p* < 0.01) groups. At 19 wpv, the CD8^+^ frequency in PBMCs was higher in animals vaccinated with QuilA®-Coxevac® compared to the Coxevac® group (Fig. 3b). This CD8^+^ population was also strongly present in splenocytes of the QuilA®-Coxevac® group at sacrifice (28.0% in QuilA-Coxevac® vs 15.9% in control and 18.3% in Coxevac®) (Fig. 4a). Interestingly, the increased frequency of CD8^+^ cells in spleens was associated with an increased frequency of a granulocytic population (3.4% in QuilA®-Coxevac® vs 1.1% in control and 1.8% in Coxevac®) (Fig. 4b). The frequency of the other sub-populations was comparable between groups in all investigated organs (Fig. 4a and Supplementary Fig. 3).

For the CD4^−^CD8^−^ cell population in PBMCs, the mixed-effects model did not identify different patterns between groups (*F*(16, 104) = 0.7; *p* = 0.7), but revealed significant differences in the average CD4^−^CD8^−^ frequency between groups post-challenge (*F*(2, 13) = 4.803; *p* < 0.02). At several time points (13, 13.5, 15, 16, 18, 19 wpv), significant pairwise differences between the Coxevac® and QuilA®-Coxevac® group were present. Additionally, at 19 wpv, the CD4^−^CD8^−^ frequency in the Coxevac® group was also significantly higher compared to control goats (Fig. 3b). To provide a deeper characterization of this CD4^−^CD8^−^ cell subpopulation, we looked at gamma-delta (γδ) T cells and WC1^+^ γδ T cells at 13.5 and 19 wpv. However, no significant differences in the relative percentage were observed between groups in these cell populations (Supplementary Fig. 4).

### Distinctive transcriptional patterns are induced in spleens and bronchial lymph nodes of vaccinated and control animals upon challenge

**Fig. 5:**
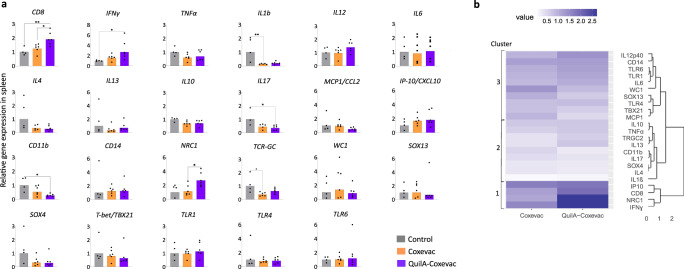
Distinctive transcriptional patterns are induced in spleens of vaccinated and control animals upon challenge. **a** Expression profiles of selected genes (*n* = 23) in control, Coxevac® and QuilA®-Coxevac® groups at sacrifice. Data are represented as scatter dot plots with a line at the geometric mean. Outliers are identified and removed using the ROUT method (*Q* = 1%). Differences between experimental groups were tested using the one-way ANOVA test with Tukey’s multiple comparison post hoc test or the Kruskal–Wallis test with Dunn’s multiple comparison post hoc test depending on the results of the homogeneity of variance and normality of residuals evaluated via the Bartlett’s and Shapiro–Wilk tests, respectively (*p ≤ 0.05; **p ≤ 0.01). **b** Hierarchical clustering heatmap analysis of data issued from the gene expression profiling in the spleen. Each colored cell on the map corresponds to the value of the geometric mean for each group. Values are measured by maximum distance with a Ward.2 clustering algorithm.

**Fig. 6:**
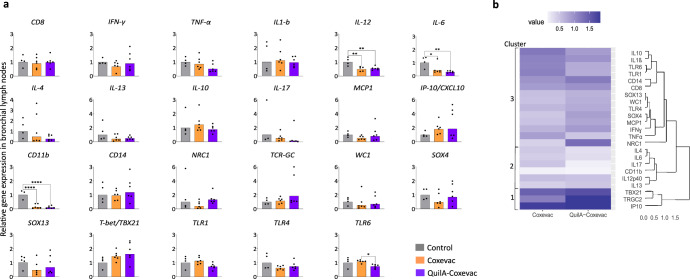
Distinctive transcriptional patterns are induced in bronchial lymph nodes of vaccinated and control animals upon challenge. **a** Expression profiles of selected genes in control, Coxevac® and QuilA®-Coxevac® groups at sacrifice. Data are represented as scatter dot plots with a line at the geometric mean. Outliers are identified and removed using the ROUT method (*Q* = 1%). Differences between experimental groups were tested using the one-way ANOVA test with Tukey’s multiple comparison post hoc test or the Kruskal–Wallis test with Dunn’s multiple comparison post hoc test depending on the results of the homogeneity of variance and normality of residuals evaluated via the Bartlett’s and Shapiro–Wilk tests, respectively (*p ≤ 0.05; **p ≤ 0.01; ****p ≤ 0.0001). **b** Hierarchical clustering heatmap analysis of data issued from the gene expression profiling in respiratory lymph nodes. Each colored cell on the map corresponds to the value of the geometric mean for each group. Values are measured by maximum distance with a Ward.2 clustering algorithm.

We next sought to further investigate the immune responses activated by the different conditions. As such, we studied the expression profiles of 23 selected genes (comprising cytokines, CDs, receptors and transcription factors) in splenocytes and bronchial lymph node cells upon infection (Figs. 5 and 6). Vaccination with QuilA®-Coxevac® skewed the transcriptional gene expression induced by infection towards an increased expression of *CD8*, *NRC1* and the Th1 cytokine *IFNγ* in spleen, corroborating the previous results of cell phenotyping and IFNγ production. In this organ, the expression of *IL1β*, *IL17A*, *CD11b*, and *TRGC2* was instead more expressed upon challenge in the control group than in the other two groups (Fig. 5a). Gene expression in the respiratory lymph nodes highlighted the differences among groups. Here, *IL12p40*, *IL6*, and *CD11b* were more expressed upon challenge in the control group than in vaccinated groups. *TLR6* was also significantly more expressed in the Coxevac® compared to the QuilA®-Coxevac® group (Fig. 6a).

To highlight specific transcriptional patterns, the relative gene expression was further used for high-level comparative analyses. In both splenocytes and bronchial lymph node cells, three different clusters were identified (Figs. 5b and 6b). The first cluster regrouped genes highly and intermediately expressed in the QuilA®-Coxevac® and Coxevac® groups, respectively. These genes included *IFNγ*, *CD8*, *NRC1*, and *IP10* in splenocytes and *TBX21*, *TRGC2*, and *IP10* in bronchial lymph nodes. The second cluster represented genes more expressed in challenged-control goats than the challenged-vaccinated groups. In splenocytes, it regrouped pro-inflammatory cytokines (*IL1β, TNFα*), Th2 cytokines (*IL4* and *IL13*), *IL17A* and *IL10*, as well as *CD11b* and *TRGC2* cellular markers and the *SOX4* transcription factor. In bronchial lymph nodes, it consisted again of *IL4*, *IL13*, *IL17A*, and *CD11b*, but included *IL6* and *IL12* as well. The third cluster comprised genes that were homogeneously expressed in all groups or for which a specific pattern was unclear.

### Specific immune responses distinguish Coxevac®, QuilA®-Coxevac® and control goats following *C. burnetii* challenge

**Fig. 7:**
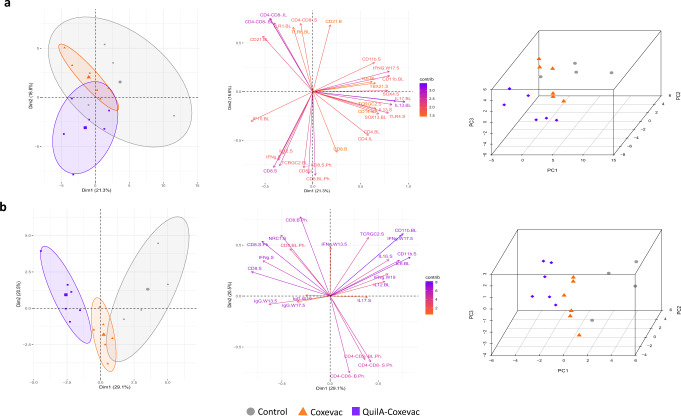
Specific immune responses distinguish Coxevac®, QuilA®-Coxevac® and control goats following *C. burnetii* challenge. **a** Principal component analysis (PCA) of the complete dataset (*n* = 68), which included data from serology (weeks 13.5, 17.5 and 19 pv), IFNγ secretion upon antigen specific stimulated PBMCs (weeks 13.5, 17.5 and 19 pv), organ and blood (week 19 pv) phenotyping and gene expression profiles. **b** PCA of specific selected variables (*n* = 22) resulting in an accurate separation of the three conditions. From the left, graph 1 is the 2D PCA score plot of the first two components (**a**, **b**). Symbols represent animals, the central one corresponds to the mean coordinates of the individuals in the group. Graph 2 is the PCA loading plot showing the distribution of all 22 variables (**b**) or of variables with a contribution more than 1.5 (*n* = 31, the loading plot containing all 68 variables is shown in Supplementary Fig. 5) (**a**). Graph 3 is the 3D PCA score plot of the first three components, realized to increase the proportion of variance illustrated by the analysis (**a**, **b**). B=blood, S=spleen, BL=bronchial lymph nodes, IL=inguinal lymph nodes, PH=phenotyping, W=week, Contrib=contribution.

In an attempt to reduce noise and extract representative information from the entire dataset to highlight immune patterns activated distinctly by vaccinated and control goats upon *C. burnetii* infection, we used the pattern recognition approach through the principal component analysis (PCA). When we used the complete dataset (*n* = 68), which includes data from serology (13.5, 17.5 and 19 wpv), IFNγ secretion upon antigen-specific stimulated PBMCs (13.5, 17.5 and 19 wpv), organ and blood (19 wpv) phenotyping and gene expression profiles, we observed that vaccinated animals from the two groups, Coxevac® and QuilA®-Coxevac®, were separated in two clusters (Fig. 7a). In these groups, even if crossing areas were present, the intra-group variance (*σ*²) was reduced compared to the control group (*σ*²_Coxevac_ = 0.71, *σ*²_QuilA-Coxevac_ = 0.99 vs *σ*²_Control_ = 1.35). Conversely, within the control group, the higher intra-group variance highlighted the different responses among animals present in this group, in particular for the C1 goat, who was the least responsive upon challenge. Overall, when considering a high level of complexity of variables, the three groups were quite close in both 2D and 3D plots (Fig. 7a). In contrast, selecting specific variables (*n* = 22) to reach distinctive clusters among conditions, characteristic features that can accurately describe each group were identified (Fig. 7b). The control group was distinguished by cytokines (i.e., *IL1β, IL12, IL6, IL17A*), CD11b^+^ cells in the secondary lymphoid organs, γδ T cells in spleen and IFNγ secretion upon antigen-specific stimulation of PBMCs. The Coxevac® group had a distinctive feature, the CD4^−^CD8^−^ lymphocyte population present in both systemic and secondary lymphoid organs. Finally, the QuilA®-Coxevac® group was distinguished by its increased IgG response, the higher frequency of CD8^+^ T cells in blood and secondary lymphoid organs, and by a greater expression of *CD8*, *NRC1*, and *IFNγ* in the spleen.

## Discussion

In the present study, we characterized the immune responses elicited by Coxevac® vaccination upon challenge of goats with *C. burnetii* and evaluated whether adjuvanting this vaccine with QuilA, an EMA-approved veterinary medicinal product, could increase its protective efficacy against Q fever. Adjuvanting vaccines with QuilA® triggers more robust humoral and cellular immune responses against a wide range of pathogens than the non-adjuvanted forms^34–37^. For the first time, an axenic culture of *C. burnetii*, isolated from a field sample, was used to produce the challenge inoculum. Before intranasal infection of goats with the CbBEC2 strain, we assessed the presence of a full-length LPS and the infectious potential of the bacterial strain in a mouse model, demonstrating the suitability of an axenic culture for the preparation of the challenge dose. Although small rodents and non-human primates have been used to study vaccine-induced protective immune responses, we decided to work with a goat model as they are natural hosts of *C. burnetii* and the target species of the Coxevax® vaccine.

In our experimental study, the humoral immunity was evaluated after vaccination and challenge in the caprine model by determining *C. burnetii*-specific IgG levels. Both Coxevac® and QuilA®-adjuvanted Coxevac® induced antigen-specific IgG, whilst only the adjuvanted form elicited a more protracted IgG response after vaccination. Upon challenge, the QuilA®-Coxevac® group mounted a stronger humoral response compared to both the challenged-control and the Coxevac® group, highlighting the enhanced effect promoted by the QuilA® in the formulation. The role of the humoral response for protection against *C. burnetii* is still undetermined in goats. In mouse models, it has been shown that the humoral response is critical for the prevention of clinical disease, but not for infection control^33^. Surprisingly, the antibody response generated by challenged control and Coxevac® goats was comparable. This suggests that the reduction of clinical symptoms (abortions) and bacterial shedding in goats as read-out of vaccine efficacy does not rely exclusively on antibodies^13–16^.

At sacrifice, live *C. burnetii* were detected in spleens and/or bronchial lymph nodes of all challenged control and Coxevac® goats, confirming that vaccination with Coxevac® was insufficient to prevent the infection^13–15^. However, *C. burnetii* DNA was under the detection limit of the PCR assay requiring bacterial sample pre-amplification in embryonated eggs. Globally, the infection was associated with a low bacterial burden in all groups. Reasons for the low infection rate could be explained by the absence of breeding in our animals^10^ or the different kinetic of infection of our strain in relation to a previous study^8^. Interestingly, vaccination with QuilA®-adjuvanted Coxevac® induced a moderate but increased protection efficacy.

Considering that production of specific antibodies was not sufficient to clear *C. burnetii* infection, we explored the vaccine-induced cell-mediated immunity. In mice, T cell-mediated immunity is critical for protection against Q fever^33,38^ and IFNγ is important for controlling bacterial replication and elimination^13,39–43^. To assess cellular *C. burnetii*-specific responses, PBMCs were ex vivo stimulated in an antigen-specific manner and IFNγ was measured upon vaccination and challenge of goats. In the Coxevac® group, a peak of IFNγ production was reached only one week after the booster dose (5 wpv). In contrast, in the QuilA®-Coxevac® group, a first peak was detected two weeks after the prime vaccination and a second peak one week after the boost (5 wpv). These results suggest that (1) the reaction time for IFNγ production was reduced to one week between the prime and the booster dose in the QuilA®-Coxevac® group and (2) the antigen-specific IFNγ response was accelerated by QuilA®-Coxevac® vaccination compared to Coxevac® only. The mechanism of action of QuilA® remains poorly understood, but the current model entails that the lipophilic moiety of Quillaja saponins promotes direct delivery of exogenous antigens to the intracellular cell compartment of antigen-presenting cells (APCs) promoting histocompatibility complex (MHC) class I presentation with consequent stimulation of CD8^+^ T cells^44^. It is possible that QuilA® favored the process of antigen uptake and presentation to promote immunomodulation towards accelerated IFNγ responses.

Upon challenge, a bimodal pattern characterized again the IFNγ response generated by antigen-stimulated PBMCs of the QuilA®-Coxevac® group, with the highest peak detected immediately after challenge (13.5 wpv) and a second peak at 17.5 and 18 wpv. Considering that goats were exposed to *C. burnetii* antigens during vaccination, the prompt response of this group as compared to control goats could be explained by a direct consequence of the vaccine prior exposure. By contrast, the IFNγ response of the Coxevac® group upon challenge was less pronounced. One hypothesis is that Coxevac® vaccination could activate different pathways or other lymphocyte subsets, promoting IFNγ-independent responses, as supported by the higher frequency of CD4^−^CD8^−^ cells detected in PBMCs of this group after challenge. The CD4^−^CD8^−^ population is involved in the control of intracellular bacteria, such as *Mycobacterium tuberculosis* or *Francisella tularensis* Live Vaccine Strain (LVS)^45^, but further investigations are needed to better characterize this population and its role in protective immunity against *C. burnetii*.

In challenged control goats, an antigen-specific IFNγ response was detected quickly upon challenge until the end of the experiment. This response was characterized by two phases: an initial moderate production promptly after challenge (13.5 wpv) followed by a sudden increase at 17–17.5 wpv (4–4.5 wpc). This second wave corresponded to the second IFNγ peak detected in the QuilA®-Coxevac® group. Four weeks could be the time needed to mount the adaptive response against *C. burnetii*, or relates to the kinetic of bacterial multiplication in vivo. Indeed, in pregnant goats, *C. burnetii* DNA has been detected in the placenta of challenged animals only 4 wpi onwards^8^. The strong IFNγ response induced in the challenged control goats by stimulated PBMCs could be the consequence of a pronounced inflammatory response. In this group, the transcriptional pattern of spleens and respiratory lymph nodes showed indeed an immune response characterized by cytokines, such as pro-inflammatory (*IL1β, TNFα, IL12*), Th2 (*IL4 and IL13*), Th17 (*IL17A*), and other immunoregulatory cytokines (*IL6, IL10*). Increased levels of *IL10*, *TNFα* and *IL1β* were previously described in peripheral blood of infected pregnant goats before or after parturition^9^. IL1β, IL6 and TNFα production were higher in naturally infected individuals compared to vaccinated ones^46^. Increased levels of inflammatory cytokines (i.e. TNF, IL6, IL12, IL10, IL1b) characterize patients with both acute and chronic Q fever^47–50^. In particular, the chronic course is related to intense inflammation, characterized by upregulation of TNF, IL1β, IL6 and excessive IL10 expression^47–49^. In addition, IL10 and IL4 support the intracellular replication of *C. burnetii* in monocytes^51,52^ and macrophages^53,54^ and the polarization of macrophages toward an M2 program^55^, proposed to be permissive for bacterial replication^56^. Trials of new candidate vaccines inducing a Th2-based response were not able to confer protection similar to Q-Vax, the licensed human vaccine against Q fever^57^, which elicits a Th1-dominant response^33,58^. Overall, IL10 and Th2 cytokines seem to interfere negatively with the control of Q fever. Thus, the overexpression of these cytokines in secondary lymphoid organs of challenged control goats may have hampered the establishment of an effective immune response able to clear *C. burnetii* in organs and to control the infection.

The immune response initiated by QuilA®-Coxevac® goats in secondary lymphoid organs was unambiguous compared to the other groups. An increased frequency of a granulocytic population was detected in spleens. In mouse models, eosinophils and neutrophils are involved in vaccine-induced protection against Q fever^59,60^. Whether a similar mechanism exists also in goats deserves further attention, but the increased frequency of splenic granulocytes in QuilA®-Coxevac® vaccinated goats seems to support this hypothesis. Interestingly, the expression level of *CD11b*, used as a marker for several cell types such as granulocytes, macrophages, NK cells, B lymphocytes and dendritic cells, was significantly increased in challenged control goats compared to the vaccinated-challenged groups. The specificity of this marker should be better investigated in goats, together with its role during *C. burnetii* infection.

The QuilA®-Coxevac® group was also characterized by an increased CD8^+^ T cell frequency in spleens and PBMCs (at 19 wpv) compared to the other groups as well as a more pronounced mRNA expression of *CD8*, *NRC1* (NK cell receptor), *IP10* and *IFN*γ, than the challenged control goats. Coxevac® vaccinated goats on the other hand presented an intermediate expression of these genes. Thus, the QuilA® adjuvant triggered a splenic Th1-type response probably driven by CD8^+^ and NK cells. This confirms observations from a previous study in mice showing that QuilA skews a Th1-type response and improves antibody levels upon immunization with *C. burnetii* phII soluble antigens^61^. Also, the protection conferred by CD8^+^ T cells results in less lung inflammation and decreased bacterial burden in spleens as compared to the protection elicited by CD4^+^ T cells, suggesting that efficacious vaccines against Q fever should elicit CD8^+^ T-cell immunity^62^. NK cells do not intervene in bacterial clearance but participate in mounting the inflammatory response against Q fever^41^. *C. burnetii* can infect NK cells, which secrete IFNγ and release lytic granules containing bacteria via degranulation^63^. Whether this also occurs in goats remains to be clarified.

The role of γδ T cells during *C. burnertii* infection or protective immunity is not characterized and some interesting data are presented here in goats for the first time. γδ T cells are distinguished by their production of IFNγ or IL17A. While the transcription factor T-bet promotes the production of IFNγ by γδ T cells, SOX4 and SOX13 promote IL17A production^64^. In bronchial lymph nodes of the QuilA®-Coxevac® group, IP10, a chemokine produced in response to IFNγ, was grouped with TBX21 (or T-bet), and TRGC2, the receptor of γδ T cells. During *C. burnetii* infection, IP10 is strongly induced by IFNγ and its expression is increased in infected mice, as a model of acute Q fever^65–67^. By contrast, in patients with chronic Q fever, IP10 expression is downregulated^68,69^. The co-clustering of IP10, TBX21 and TRGC2 genes suggests the involvement of IFNγ-producing γδ T cells after challenge in this group. T-bet has been described to play a key role during *C. burnetii* primary and protective immunity. Tbet^−/−^ mice experience more severe infection and a decreased ability to control bacterial replication not only after primary infection but also after vaccination-challenge^60,70^. By contrast, primary infection of goats was identified with a rise of SOX4/IL-17A and γδ T cell receptor transcripts, highlighting a different role for γδ T cell subpopulations during vaccination and infection. During acute Q fever infection, circulating γδ T cells increase in patients^71^. In vitro studies show that *C. burnetii* downregulates the IL-17A signaling pathway in murine alveolar macrophages and that the activation of this pathway results in bacterial elimination^72^. On the other hand, Elliott et al.^59^ demonstrated that IL17A is not essential during Q fever infection. To date, the exact role of IL-17A producing γδ T cells in the immune response activated after *C. burnetii* infection remains to be elucidated. While in our study different γδ T cell transcript profiles were present in vaccinated vs control animals, no changes in the relative frequency of γδ T cells and WC1^+^ γδ T were observed. This suggests that possible effects on γδ T cells due to vaccination or challenge could relate to changes in their functional role rather than their relative abundance.

To have a global view of the immune responses activated by the different conditions, we used a principal component analysis. This analysis corroborated the existence of three distinct clusters and suggests that the activation of a Th1-based response by QuilA®-Coxevac® vaccination helps the clearance of infection. Down-regulation of these pathways would be used as survival strategy by the bacterium, as observed in challenged control goats, which presented a different immune response. The transient increase of the rectal temperature observed after QuilA®-Coxevac® prime vaccination might reduce the acceptance of the vaccine. This could be overcome by reducing the antigen level or using other adjuvants promoting, as QuilA, Th1 responses.

In conclusion, this study described for the first time the immune response generated by Coxevac® vaccination in a goat model of aerosol challenge with an axenic culture of *C. burnetii* isolated from a field sample. Coxevac® did not induce a significant humoral or cellular response able to confer substantial protection upon challenge. It confirmed previous field studies and presented insights associated with the low immunogenicity of this vaccine and the need to improve its formulation, including reconsidering several aspects such as the antigen, the dose or the inactivation method. Seeing that the addition of QuilA® to the Coxevac® formulation increased the humoral response and elicited a sustained Th1-type cellular response, which improved vaccine efficacy, we propose to test the use of QuilA® to increase Coxevac® efficacy against *C. burnetii* infection in field settings.

## Methods

### *C. burnetii* strain and culture

CbBEC2 is a Belgian strain representative of the SNP2 genomic group, isolated from infected caprine milk. The strain, initially isolated in vivo in BALB/c mice^73^, was either (1) propagated again in BALB/c mice (Charles Rivers, Wilmington, MA, USA) (Passage P2), from now on indicated as freshly collected splenic harvest, or (2) propagated in African green monkey kidney (Vero) cells (2 passages) and then in ACCM-2^74,75^ (3 passages) (P6). The CbBEC2 strain was isolated and maintained at the National Reference Laboratory for *Coxiella burnetii* and *Bartonella* (Sciensano, Belgium).

### Analysis of *C. burnetii* LPS

The presence of a full-length LPS, distinctive of virulent phI strains, was assessed in the CbBEC2 strain by WGS and SDS-PAGE silver staining. Genomic DNA extracted from a 14-day ACCM-2 culture (P6) was sequenced on the Illumina MiSeq platform and assembled as described^76^. To ascertain the presence of genes involved in the synthesis of a full length LPS, CbBEC2 assembly was aligned in NCBI BLASTN (Galaxy version 2.10.1) with the NM phI sequence of the operon involved in the biosynthesis of the complete LPS (Genbank accession number: AF387640). Illustration of the alignment was obtained using the CLC sequence viewer software (Version 8.0).

*C. burnetii* LPS from confluent ACCM-2 cultures was extracted as previously reported^29^. LPS (6 µl, diluted 1:3) was then electrophoresed (120 V, ±1 h) on 4–20% precast protein gels (Biorad, Hercules, CA, USA) together with the PageRuler prestained protein ladder (ThermoFisher Scientific, Waltham, MA, USA). The gel was fixed for 1 h in a solution composed of 25% (vol/vol) ethanol, 8% (vol/vol) acetic acid, 25% (vol/vol) formaldehyde solution (37 wt. % in H_2_O) and washed trice, 20 min each, with 50% (vol/vol) ethanol solution.LPS was visualized using silver staining of the gel by consecutive baths of 0.8 mM sodium thiosulfate pentahydrate (1 min), 10 nM silver nitrate (20 min in the dark), 250 nM sodium carbonate (2.5 min in the dark) and 25 nM Na_2_EDTA.2H_2_O (10 min). Gels were imaged using a digital camera.

### Analysis of the *C. burnetii* infectious potential in a mouse infection model

Six-week-old female BALB/c mice were injected intraperitoneally with 4–5 × 10^3^ CbBEC2 *C. burnetii* cells (200 µl) derived from freshly collected splenic harvest (P2) or a 4-day old axenic ACCM-2 culture (P6). The axenic culture was arrested at the exponential phase of bacterial growth. Dilutions of *C. burnetii* from stock suspensions were obtained in 0.9% saline solution (Biorad, Hercules, CA, USA). Control mice were injected with 0.9% saline solution only. Inocula were quantified with the monocopy *com1* real-time PCR^77^. The cycle threshold (Ct) was used to calculate the genomic equivalents (G.E.) of the suspension pondered against a calibration curve obtained with an external reference (ThermoFisher Scientific, Waltham, MA, USA). A group of six mice was used for each strain type and for the four time-points (48 mice in total). At sacrifice, serum and organs were collected from each animal to evaluate the serological responses (IgM and IgG), the organ weight (spleen and liver) and the bacterial load (G.E. in spleen) as described previously^77^.

### Vaccination-challenge experimental protocol in goats

Sixteen 2–3 years old female Saanen goats from the same Q fever free herd (defined as free of disease in the past 8 years by the bulk tank milk surveillance program) were tested for Q fever, brucellosis, *Chlamydia abortus*, paratuberculosis, Schmallenberg virus, and caprine arthritis encephalitis virus. Assuming a medium level of difference between conditions (effect size = 0.25 using the *F* test^78^), a power analysis was used to calculate the required number of animals in each group. Goats were randomly distributed into three experimental groups: control (*n* = 4), vaccine only (Coxevac® group, *n* = 6) and vaccine plus adjuvant (QuilA®-Coxevac® group, *n* = 6). Animals were allocated to three independent, separated, BSL-2 housing boxes of 19 m^2^ floor area (3.2 m^2^/animal), complying with the minimum requirements established by the Belgian law for housing goats of any weight. Prior to challenge, goats were transferred to the BSL-3 area and the animals were housed in three separated boxes of 18 m^2^ floor area (3 m^2^/animal), under a common filtered-ventilated area. Each housing box was separated by one empty box. Both in the BSL-2 and BSL-3 facilities, circadian rhythm was maintained with artificial light. Feeding was *ad libitum* and cleaning of the boxes occurred daily. Figure 1a shows the timeline of the vaccination-challenge experiment. After 2 weeks of acclimatization, goats were vaccinated subcutaneously 2 cm above the *Spina scapula* with 2 ml of Coxevac® (72 QF Units/ml, CEVA Sante Animale, Libourne, France) or with 150 μg of QuilA® (Invivogen, San Diego, CA, USA) diluted in 2 ml of Coxevac®. As controls, goats were injected with 2 ml of PBS (pH 7.4, Gibco). At 4 wpv, goats received a booster dose following the manufacturer’s recommendations.

At 13 wpv, all groups received an intranasal challenge with 10^6^ CbBEC2 *C. burnetii* derived from 4-day old axenic ACCM-2 culture (P6). Inocula were diluted in 1 ml PBS and quantified with the monocopy *com1* real-time PCR. Inoculation (0.5 ml/nostril) was performed using a small nebulizer with 1-mm spray opening (Boehringer Ingelheim, Ingelheim, Germany) fixed on a syringe. Nostrils were held alternately closed during inhalation.

The general health and the rectal temperature of goats were monitored daily by clinical inspections until the end of the experiment (19 wpv). Blood was collected for serological and immunological analyses at the indicated time points (Fig. 1a). At the end of the study, goats were sacrificed by stunning with a penetrating captive bolt followed by immediate exsanguination. Blood and organs were collected for bacterial detection, cell phenotyping and gene expression profiling.

### *C. burnetii*-specific antibody response

Total IgG antibody levels directed against *C. burnetii* (antigen phI + phII) in goats were measured using the commercially available PrioCHECK^TM^ Ruminant Q fever Ab Plate Kit (Applied Biosystems, Thermo Fisher Scientific Inc.) according to the manufacturer’s instructions. Briefly, serum samples (diluted 1:400) were added to the plate coated with *C. burnetii* whole-cell antigens and detected with the HRP-conjugated G protein at 1:100 dilution. The optical densities (OD) were measured at dual wavelengths of 450–620 nm on a microplate reader (Sunrise, Tecan Trading AG, Switzerland). S/P% >40 was considered as positive.

### IFNγ ELISA

Blood was sampled from goat jugular vein on BD Vacutainer® Heparin Tubes (BD Bioscience, San Jose, CA, USA) and PBMCs were isolated using Ficoll®-Paque PREMIUM 1.073 (GE Healthcare, Chicago, IL, USA) density gradient centrifugation. After washing, PBMCs were resuspended in RPMI 1640 Medium with GlutaMAX™ (Gibco) and 10% fetal bovine serum (FBS) (Gibco) as supplement. Cells were seeded in 180 uL at 3.0 × 10^5^ cells/well in 96 well-plates (VWR, Radnor, PA, USA) and incubated either with 20 µl 1× PBS, *C. burnetii* CbBEC2 inactivated antigen (2 × 10^7^/ml) or pokeweed mitogen (PWM) at 5 μg/ml (positive control) for 48 h. After centrifugation, the supernatant was harvested and stored at −20 °C. IFNγ secretion was measured in the supernatant with a sandwich ELISA using the ID screen® Ruminant IFNγ kit (IDvet, Grabels, France) according to the manufacturer’s instructions. Antigen-specific stimulation was validated for each time point by the positive (PWM) and negative (PBS) controls of stimulation. Recombinant goat IFNγ (Cusabio, Houston, TX, USA) was used to validate the recognition of goat IFNγ by the antibodies provided in the kit.

### Isolation of splenocytes and lymph node cells

At sacrifice, caprine spleens, bronchial and inguinal lymph nodes were aseptically removed and transported to the laboratory in RPMI 1640 medium with GlutaMAX™ (Gibco) and 10% FBS (Gibco). One cm^3^ of tissue was disrupted and homogenized in the transport medium for cell isolation. After passage through 70 µM cell strainers (Corning Life Science, Corning, NY, USA), cells were centrifuged (1200 rpm, 10 min) and resuspended in RPMI 1640 medium with GlutaMAX™ (Gibco) and 10% FBS (Gibco) as supplement. Splenocytes were incubated with the BD Pharm Lyse™ lysing solution (BD Bioscience, San Jose, CA, USA), according to the manufacturer’s protocol, for red blood cell lysis. Successively, splenocytes, bronchial and inguinal lymph node cells were immediately stained for cell phenotyping or cryopreserved in 90% FBS (Gibco) with 10% DMSO (Merck, Darmstadt, Germany) for further testing (RNA extraction or *C. burnetii* detection).

### Immunophenotyping with flow cytometry

PBMCs, splenocytes, bronchial and inguinal lymph node cells isolated from goats were immediately (ex vivo) stained for phenotyping in 50 µl of PBS containing a mix of monoclonal antibodies (mAb) against CD4 (Alexa Fluor®647, clone 44.38, MCA2213A647, 1:320 dilution, Bio-rad), CD8 (FITC, clone 38.65, MCA2216F, 1:320 dilution, Bio-rad) and CD21 (RPE, clone CC21, MCA1424PE, 1:10 dilution, Bio-rad). Mouse IgG1-RPE (MCA928PE, 1:10 dilution, Bio-rad) was used as isotype control. The LIVE/DEAD™ Fixable Near-IR Dead Cell Stain Kit (Invitrogen, Waltham, MA, USA), diluted 1:1000 with PBS, was used to stain dead cells. Several vials of PBMCs cryopreserved in 90% FBS (Gibco) with 10% DMSO (Merck, Darmstadt, Germany) were thawed following standard procedures and stained in 50 µl of PBS containing mAb anti-WC1 (Clone CC15, MCA838GA, 1:200 dilution, Bio-rad) or anti-γδ TCR1-N24 δ chain specific (G-GB21A, G-BOV2058, 1:200 dilution, Monoclonal Antibody Centre, Washington State University, Pullman, WA, USA) and the secondary antibody rabbit anti-mouse IgG-RPE (STAR12A, 1:20 dilution, Biorad). The LIVE/DEAD™ Fixable Aqua Dead Cell Stain Kit (Invitrogen), diluted 1:1000 in PBS, determined the viability of cells. The staining procedure required a 30 min incubation at 4 °C in the dark. After washing in PBS, cells were preserved in 4% PFA diluted in PBS (Santa Cruz Biotechnology, Dallas, TX, USA) (max. overnight) until flow cytometry was performed with BD FACSVerse™ Cytometer (BD Bioscience, San Jose, CA, USA). Data acquisition and analysis were accomplished using BD FACSuite (BD Bioscience) and FlowJo software (FlowJo, LLC, Ashland, OR, USA), respectively.

### Gene expression analysis

RNA was isolated from 5 × 10^6^ cryopreserved splenocytes and bronchial lymph node cells of goats using the RNeasy Mini Kit (Qiagen, Hilden, Germany), following the manufacturer’s guidelines. DNA contamination was removed by treatment with the RNase-Free DNase Set (Qiagen). The concentration and purity of RNA samples was assessed using Nanodrop 1000 (Isogen Life Science, De Meern, The Netherlands) and the reverse transcription was performed on equal RNA amounts for each sample with the QuantiTect Reverse Transcription Kit (Qiagen). Gene expression was quantified by real-time PCR using the LightCycler^®^ 480 SYBR Green I Master (Roche, Basel, Switzerland) following the manufacturer’s recommendations in a Light cycler® 480 Instrument II (Roche) with cycling conditions of pre-heat (10 min at 95 °C), denaturation (40 times 15 s at 95 °C), annealing (40 times 1 min at 60 °C). Primers (Supplementary Table 3) were designed on the NCBI Primer-Blast tool (https://www.ncbi.nlm.nih.gov/tools/primer-blast/) and PCR efficiency was defined for all genes using the standard curve method. Six genes (*18S, GAPDH, HMBS, HSP90, SDHA, YWHAZ*,) were selected as possible reference genes according to the literature^9,11,79–83^. Three genes (*GAPDH, HMBS, YWHAZ* for splenocytes and *GAPDH*, *HMBS, HSP90* for bronchial lymph node cells) were selected as the most stable reference genes using geNorm NormFinder^84^ and Bestkeeper^85^ software packages. The relative gene expression was calculated using the model described in Hellemans et al.^86^, which takes into account primer efficiency and three (or more) reference genes.

### *C. burnetii* detection

The presence of *C. burnetii* in goats was investigated in blood (post-challenge) and organs listed in Supplementary Table 1. Briefly, DNA was extracted from 200 µl of blood or homogenized tissues (max. 1 g in 1 ml MilliQ water) with the MagMax™ Isolation Kit (Applied Biosystems; Thermo Fisher Scientific, Inc.) according to the manufacturer’s instructions. *C. burnetii* DNA was detected on a 7500 Real-Time PCR System (Applied Biosystems, Thermo Fisher Scientific Inc.) by real-time PCR targeting the IS*1111* repetitive element as previously described^76^.

Considering that the detection limit of the PCR assay was reached, a volume of 200 µl containing approximately 5 × 10^5^ frozen splenocytes and bronchial lymph nodes cells, suspended in 1× PBS (pH 7.4, Gibco), were injected into embryonated eggs (triplicates for each sample) for bacterial amplification as described^73^. The yolk sac was harvested and homogenized in 9 ml 0.9% saline solution. The samples (200 µl) were inactivated for 40 min at 80 °C and incubated for 30 min at 37 °C with 180 µl of lysis buffer (20 mM Tris pH8, 2 mM EDTA, 1.2% Triton x-100) containing 20 mg/ml lysozyme (Roche) and 4 µl of RNase A (17,500 U) (Qiagen). DNA was extracted using the QIAGEN kit DNeasy (Qiagen) according to the manufacturer’s guidelines. The real-time PCR was run as described before in a Light cycler® 480 Instrument II (Roche). The cut-off for positivity was set at a Ct-value <40 (accredited validation file). Goats presenting at least one positive result were considered to be positive.

### Data analysis

General statistical analyses were performed as indicated in figure legends using GraphPad Prism Software Version 9 (San Diego, CA, USA). A *p* value ≤0.05 was considered significant.

The clustering analysis was performed in R using data issued from the gene expression profiling. Briefly, the distance matrix was calculated applying the maximum distance, the hierarchical clustering was run with the hclust function using the ward.D2 method and the dendrogram was plotted employing the ggdendro package. The heatmap was created via the ggplot2 package after data transformation with tidyverse package. Finally, the multi plot figure was constructed using the grid package.

The PCA was computed in R using the prcomp function on selected data. PCA results were extracted and visualized using the factoextra package, while the 3D visualization was performed with the scatterplot3D package.

### Reporting summary

Further information on research design is available in the Nature Research Reporting Summary linked to this article.

## Supplementary information

Supplementary Info

REPORTING SUMMARY

## Data Availability

All data generated or analyzed during this study are included in this published article and its Supplementary Information files.
