# Supplementary material for: QuilA® adjuvanted Coxevac® sustains Th1-CD8+-type immunity and increases protection in Coxiella burnetii-challenged goats
Source: NPJ Vaccines. 2023 Feb 14;8:17. doi: 10.1038/s41541-023-00607-z (PMC9929268; doi:10.1038/s41541-023-00607-z)
Supplement: Supplementary file 1 — Supplementary Info [file 41541_2023_607_MOESM1_ESM.pdf]

SUPPLEMENTARY DATA

Supplementary Table 1. List of organs and body fluids investigated for *C. burnetii* presence.

**Respiratory tract**	**Liver**
Pulmonary lymph nodes	Lymph node
Mucosa	Parenchyma
Tonsils	**Alimentary tract**
Bronchia	Mesenteric lymph nodes
Lung	Colon
**Female genital tract**	**Urinary tract**
Inguinal lymph nodes	Kidney
Udder	Suprarenal glands
Teat	Perineal fat
Ovaries	Bladder
Cervical mucus	Urine
Vaginal mucus	**Other**
**Hematopoietic system**	Cerebrospinal fluid
Spleen	Brain
Bone marrow	Mitral and tricuspid heart valve
Blood	Glandula parotis
	Milk

Supplementary Table 2. *C. burnetii* DNA detection in blood of control, Coxevac® and QuilA®-Coxevac® groups at selected time points post challenge.

Days post challenge	Control				Coxevac						QuilA-Coxevac					
	1	2	3	4	1	2	3	4	5	6	1	2	3	4	5	6
**1**	-	-	+	-	+	-	+	+	+	+	+	+	+	-	-	+
**3**	-	-	-	-	-	+	-	-	-	-	-	-	-	-	-	-
**7**	-	-	-	-	-	-	-	-	-	-	-	-	-	-	-	-
**10**	+	-	-	-	-	-	-	-	-	-	-	-	-	-	+	-
**14**	-	-	-	-	-	-	-	-	-	-	-	-	-	-	-	-
**17**	-	-	-	-	-	-	-	-	-	-	-	-	-	-	-	-
**21**	-	-	-	-	-	-	-	-	-	+	-	-	-	-	-	-
**35**	-	-	-	-	-	-	-	-	-	-	-	-	-	-	-	-
**42**	-	-	-	-	-	-	-	-	-	-	-	-	-	-	-	-

- : Negative Real-Time PCR

\+ : Positive Real-Time PCR

Supplementary Table 3. Primer sequences of candidate reference and target genes used in the gene expression analysis.

Gene	Full name	GenBank accession number*	Forward primer (5'-3')	Reverse primer (5'-3')	Concentration (nM)	Reference
18S	18S ribosomal RNA	DQ149973.1	TAATCCCGCCGAACCCCATT	GGTGTGTACAAAGGGCAGG	200	Sahu et al. (2018)
GADPH	Glyceraldehyde-3-Phosphate Dehydrogenase	AF017079	GCAAGTTCCACGGCACAGTC	CCCACTTGATGTTGGCAGGA	200	Zhang et al. (2013)
HMBS	Hydroxymethylbilane Synthase	XM_012095569.2	CTTGCCAGAGAAGAGTGTGG	CAGCCGTGTGTTGAGGTTTC	200	Sahu et al. (2018)
HSP90	Heat Shock Protein 90 kDa	XM_005696358.2	GCCTGCTCGGTGTTCTCTAAT	AGCGAATCTTGTCCAAGGCATCA	200	This study
SHDA	Succinate Dehydrogenase complex subunit A	XM_018065656.1	TGTCTTCTTGCCAGCGAAGC	CGCGGAGAGACAGACCTAGT	200	This study
YWHAZ	Tyrosine 3-Monooxygenase/Tryptophan 5-Monooxygenase Activation Protein Zeta	NC019466	CTGAACTCCCCTGAGAAAGC	CTGCTTCAGCTTCGTCTCCT	200	Modesto et al. (2013)
CD11B (ITGAM)	Cluster of Differentiation Molecule 11B	XM_018040558.1	ACAAGCTACTTCTCAAGGCCAA	CACTGGACATGTTCTGGTGAC	200	This study
CD14	CD14 Molecule	NM_001361647.1	TCGTCTCAAGGAACTGACGC	CACGTTACGGAGACTGAGGG	400	This study
CD8A	CD8a Molecule	XM_018055353.1	CATCTGCTACCGCCGGAAC	TTGTAGTGGCTGTCACACGG	200	This study
IFNG	Interferon Gamma	NM_001285682.1	GCTGATTCAAATTCCGGTGGA	CTCCGGCCTCGAAAGAGATT	200	This study
IL10	Interleukin 10	XM_005690416.3	ACATCAAGGAGCACGTGAACT	ACCCCTCTCTTGGAGCATATTGA	200	This study
IL12B (IL12P40)	Interleukin 12B	AF007576.1	AACCAGACCCACCCAAGAAC	GTGGCATGTGACTTTGGCTG	200	This study
IL13	Interleukin 13	XM_005682617.2	GCAGACCCTGACCACCTAAG	AGGCATCACAGGCTCAAGTC	200	This study
IL17A	Interleukin 17A	NM_001285725.1	CACAGCGAGCACAAGTTCAT	TCAGAAGCAGTAGCAGTGACATA	200	This study
IL1B	Interleukin 1 Beta	XM_013967700.2	CTCCAGCCAACCTTCATTGC	GTTGGGTGCAGCTCTTCATCT	200	This study
IL4	Interleukin 4	NM_001285681.1	GACGTCTTTGCTGCCCCA	GCTTCATTCACAGAACAGGTCTTGC	200	This study
IL6	Interleukin 6	NM_001285640.1	AATCTGGGTTCAATCAGGCGA	GCTCTGCAACTCCATGACAG	200	This study

IP10 (CXCL10)	Interferon Gamma-Induced Protein 10 KDa	NM_001285721.1	AAGTCCCAGCCTTGCTACTG	CTTGGAGGGATGGCAGTGAA	200	This study
MCP1	Monocyte Chemotactic protein-1	XM_005693218.3	CGCTGCAACATGAAGGTCTC	TTGGGAGTTAATTGCATCTGGC	200	This study
NRC1	Natural Cytotoxicity Triggering Receptor 1	NM_001285742.1	GGTGCTTTGGCTCCTACAAC	TCGTAGGAATCCCAGTAGTCAGA	200	This study
SOX13	SRY-Box Transcription Factor 13	XM_018060001.1	AGCCTTAGACACTTCCCCCA	CATGGCTTCTTCCAGTGGGT	400	This study
SOX4	SRY-Box Transcription Factor 4	XM_018039041.1	ATCCCTTTCATTCGGGAGGC	GTTGCCGGACTTCACCTTCT	400	This study
TBX21	T-Box Transcription Factor 21	XM_018064958.1	AAGCAGGGACGACGGATGT	CCACTTGCCGCTCTGATACC	900	This study
TCRGC2	T-cell Receptor Gamma-2 chain C region	XM_018047197.1	TGGAAGGTGAAAACAGTGCCC	AGTTGACCTCTCGGTAGCCA	200	This study
TNFα	Tumor Necrosis Factor Alpha	NM_001286442.1	CCAGAGGGAAGAGCAGTCC	GGCTACAACGTGGGCTACC	200	Puech et al. (2015)
TRL1	Toll Like Receptor 1	NM_001285605.1	ATCCTGATCTTGCTGGAACCC	TCTGCTGCTTTTCCCATCAGTT	400	This study
TRL4	Toll Like Receptor 4	NM_001285574.1	CCTTGCGTCCAGGTTGTTCC	AGCATCTCGGTTGATACGGG	200	This study
TRL6	Toll Like Receptor 6	NM_001285540.1	TCCAATGTTCCTGTGCGCTA	ACGTATAAGAAGGCCAGCCC	200	This study
WC1	Antigen WC1.1-like	XM_018048530.1	GTGAAGCACCAAGGAGAATGG	ACACTCCGTGATAGCTGACTC	200	This study

*Last accessed on 27/11/2022

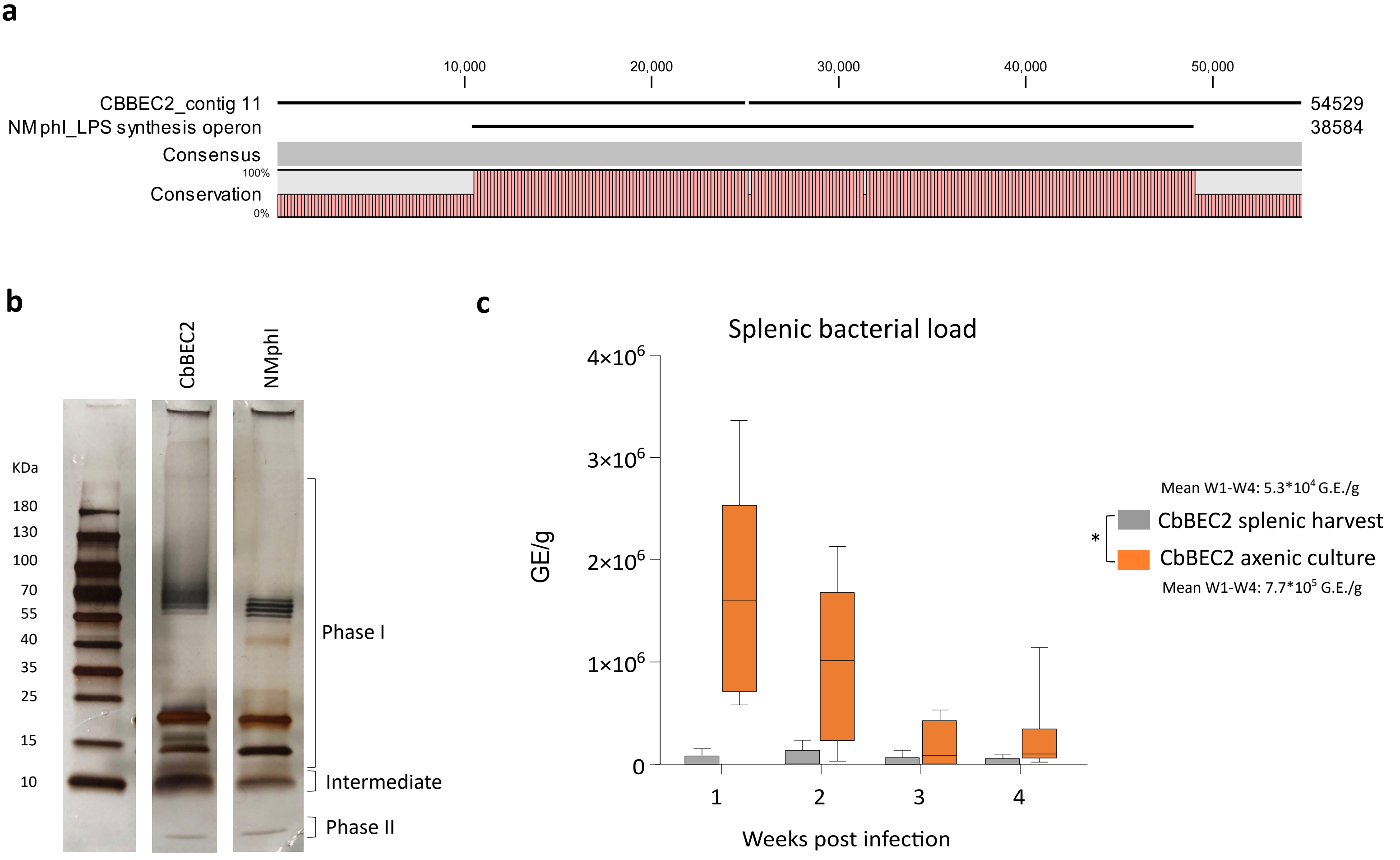

Supplementary Figure 1. *In vitro* and *in vivo* (mice) quality controls of the bacterial inoculum. **(a)** *C. burnetii* CbBEC2 strain bears phase I (phI) LPS as shown by the alignment of CbBEC2 contig 11 (54529 bp), extracted from the CbBEC2 assembly, and the NM phI sequence of the operon involved in the biosynthesis of the complete LPS (38584 bp). Consensus (in grey) represents the consensus sequence resulting from the alignment. Conservation (in red) shows the conservation level for each position in the alignment. The height of the bar displays the percentage of conservation for each position that in this case can oscillate between 50% (the position is present in one input sequence) and 100% (the position is present in both input sequences).**(b)** SDS-PAGE silver staining of CbBEC2 LPS at the cumulative passage 6 (P6) cultivated for 14 days in ACCM-2 and Nine Mile phI LPS **(c)** Comparison of the proliferative capacity of CbBEC2 strain from freshly collected splenic harvest at P2 and that from the axenic culture at P6 in the Balb/c model at 4 time points post infection. The axenic culture triggered significantly higher bacterial loads than the splenic harvest P2 in the spleen when comparing the mean of the 4 time points for each group using the Mann Whitney test (*$P \leq 0.05$). Data are represented as boxplots extended from the 25th to 75th percentiles, with a line at the median, and whiskers go from minima to maxima. GE/g = Genome Equivalents/ gram, W= week after infection.

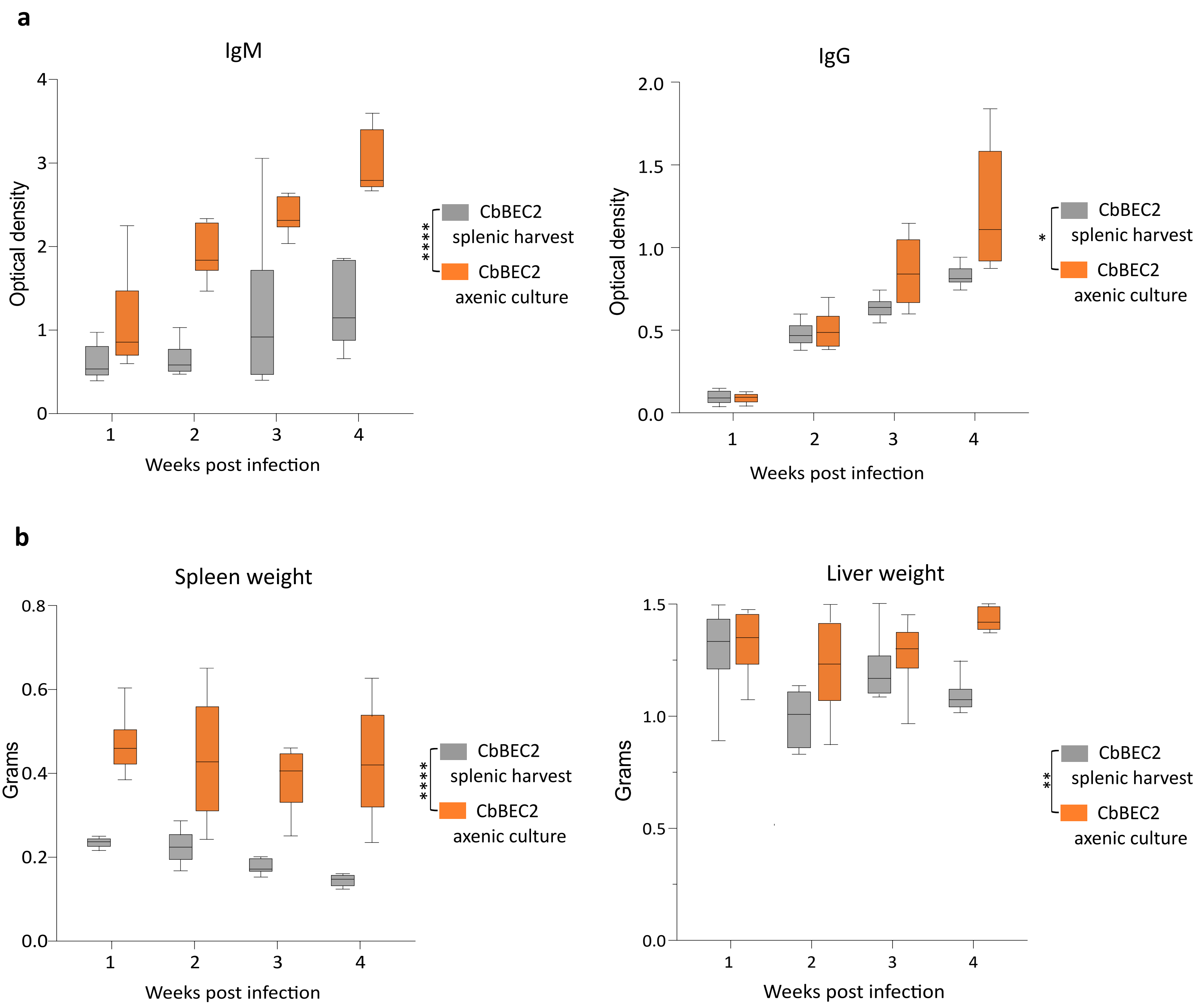

Supplementary Figure 2. Comparison of the proliferative capacity of CbBEC2 strain from freshly collected splenic harvest at P2 and that from the axenic culture at P6 in the Balb/c model at 4 time points post infection. The axenic culture triggered significantly higher IgM and IgG serum levels **(a)** and significantly higher organ weights in the spleen and the liver **(b)** when comparing the mean of the 4 time points for each group using the Mann Whitney test (*P ≤ 0.05; **P ≤ 0.01; ***P ≤ 0.001, ****P ≤ 0.0001). Data are represented as boxplots extended from the 25th to 75th percentiles, with a line at the median, and whiskers go from minima to maxima.

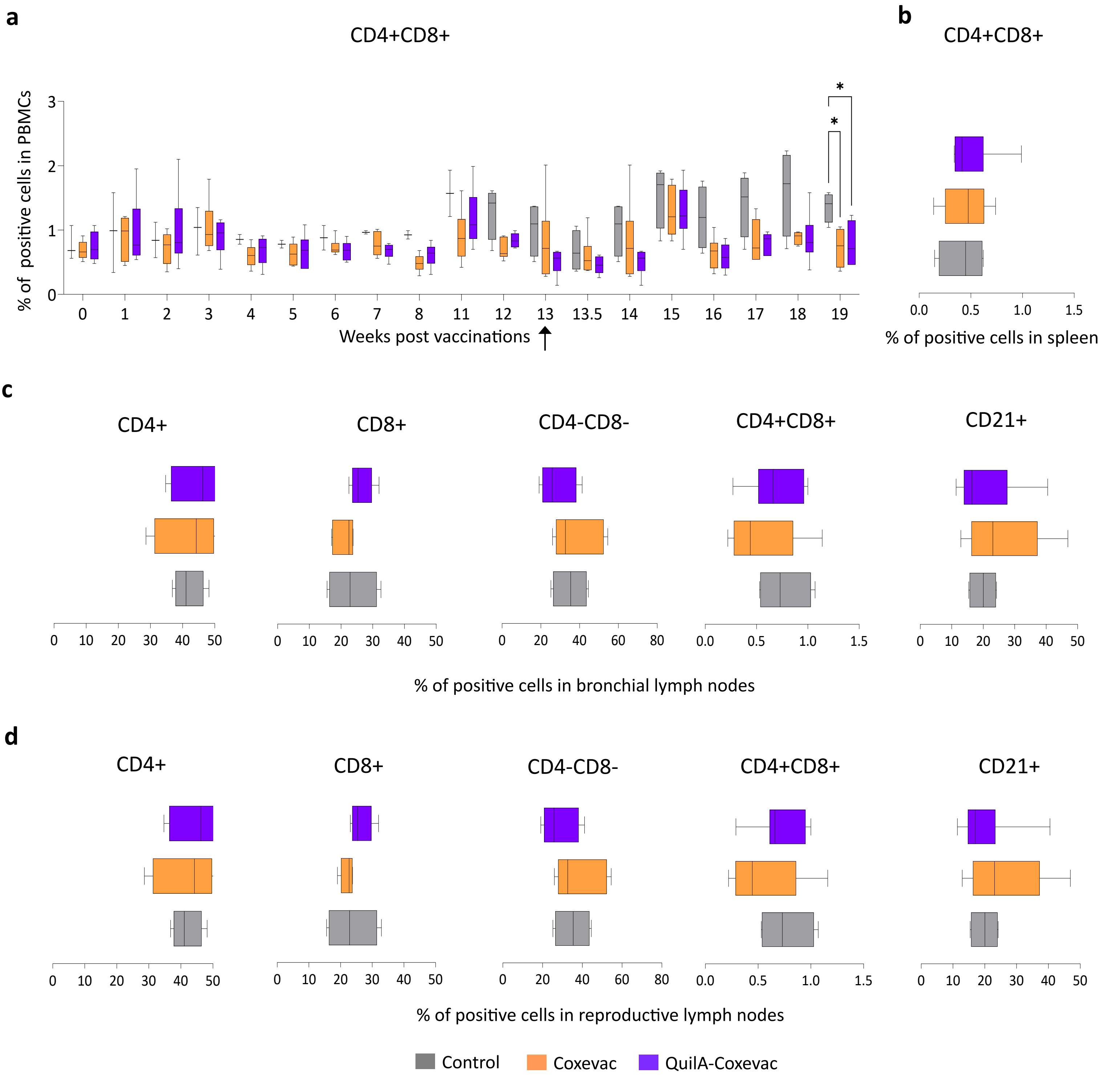

Supplementary Figure 3. (a) Kinetics of CD4+CD8+ cell frequencies in PBMCs upon vaccination and challenge. The kinetic response was analyzed using the mixed-effects models, both after vaccination and challenge, with the Geisser-Greenhouse correction followed by Tukey's multiple comparison post-hoc test. **(b)** CD4+CD8+ cell frequencies in spleens of the control, Coxevac and QuilA-Coxevac groups at sacrifice. **(c-d)** Frequencies of CD4+, CD8+, CD4-CD8-, CD4+CD8+ and CD21+ cells in bronchial and reproductive lymph nodes at sacrifice. Group comparisons were performed using One way ANOVA test with Tukey's multiple comparison post-hoc test (no statistically-significant differences were detected between groups) (b-d). The gating strategy is shown in Figure 5A (a-d). Cell frequencies are the percent of the viable cell population and data are represented as boxplots extended from the 25th to 75th percentiles, with a line at the median, and whiskers go from minima to maxima (a-d). ↑= Moment of challenge.

a

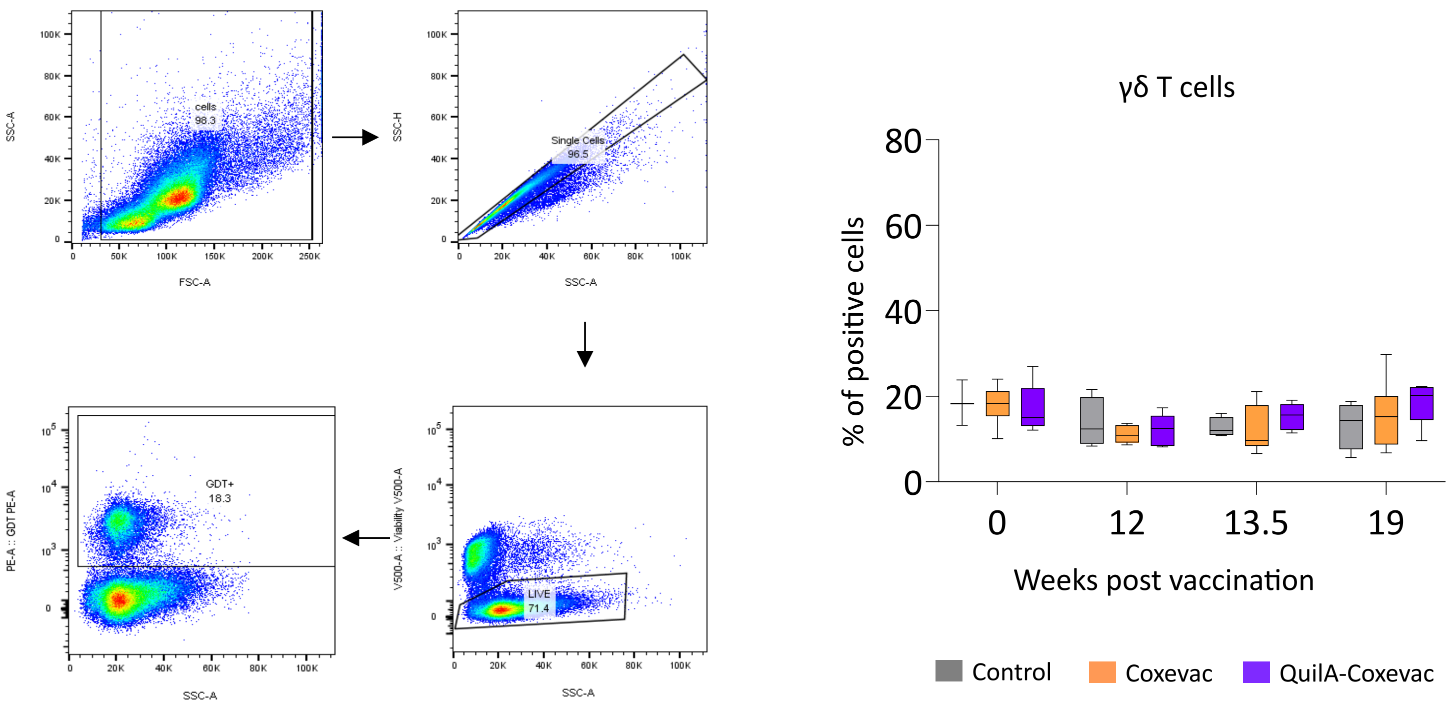

b

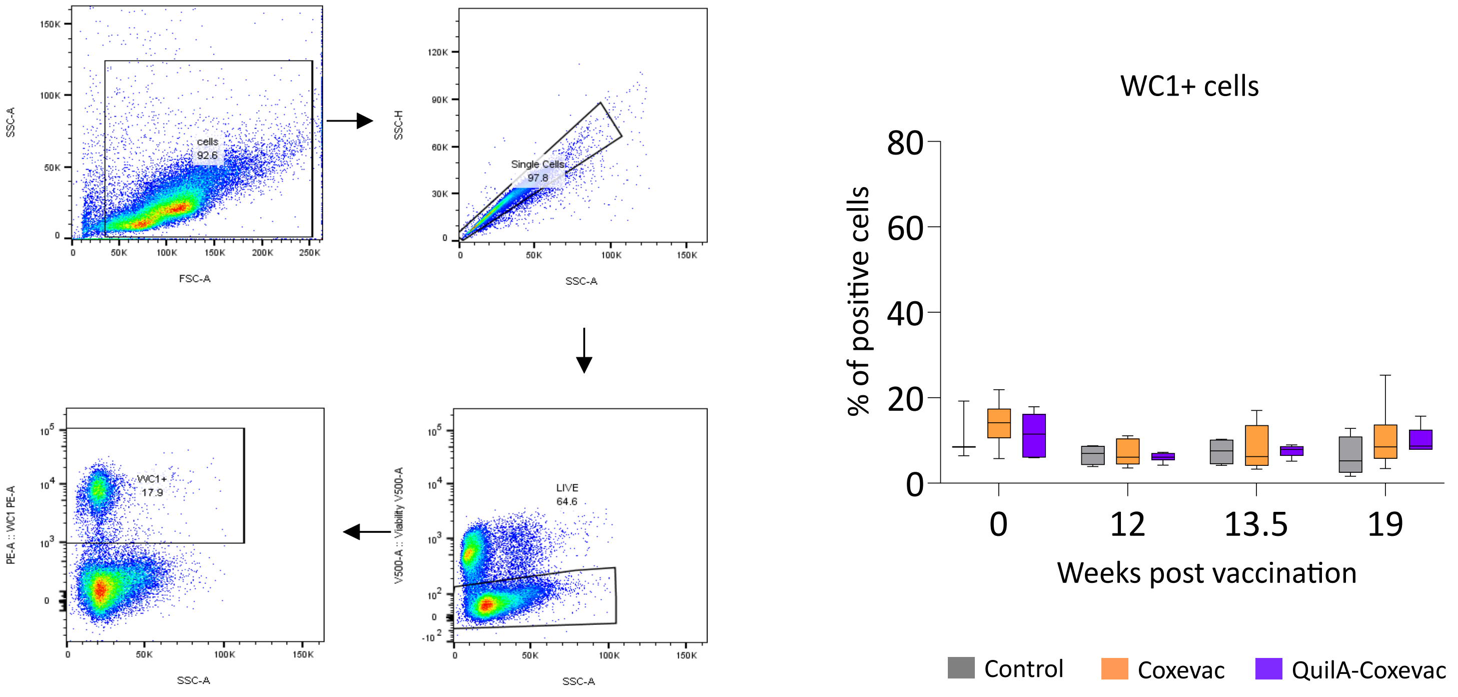

Supplementary Figure 4. Gating strategy and frequencies of γδ T cells **(a)** and WC1+ γδ T cells **(b)** in PBMCs at weeks 0, 12, 13,5 and 19 pv. Cellular subtypes were identified based on the expression of TCR1-N24 δ chain specific and WC1 cell markers. Cell frequencies are the percent of the viable cell populations. Data are represented as boxplots extended from the 25th to 75th percentiles, with a line at the median, and whiskers go from minima to maxima. Group comparisons were performed using the One way ANOVA test with Tukey's multiple comparison post-hoc test (no statistically significant differences were detected between groups).

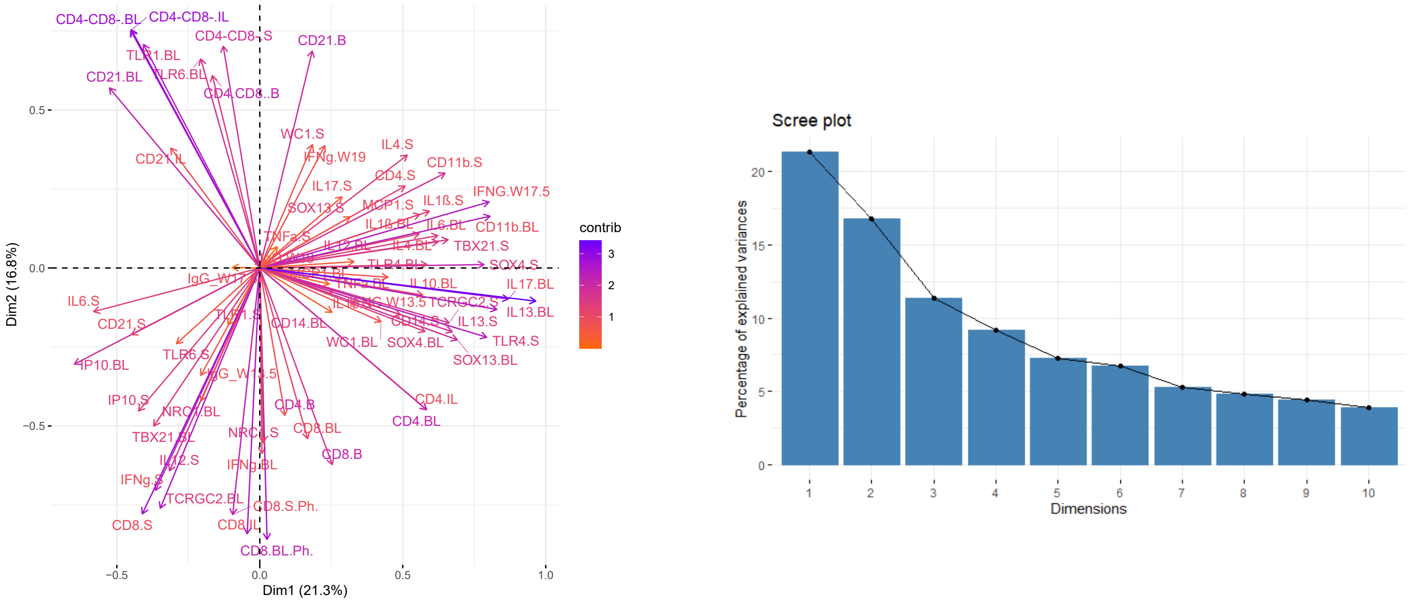

Supplementry Figure 5. Principal component analysis (PCA) of the complete dataset (n = 68), which includes data from serology (weeks 13.5, 17.5 and 19 pv), IFNƔ secretion upon antigen specific stimulated PBMCs (weeks 13.5, 17.5 and 19 pv), organ and blood (week 19 pv) phenotyping and gene expression profiles. From the left, graph 1 is the PCA loading plot showing the distribution of all 68 variables. Graph 2 is the scree plot displaying the percentage of explained variation of each principal component captured from the dataset. The corresponding PCA score plot is showed in Figure 9A. B = blood, S = spleen, BL= bronchial lymph nodes, IL= inguinal lymph nodes, PH = phenotyping, W= week, Contrib = contribution.
